# Psychological Factors in Disease

**Published:** 1938

**Authors:** T. M. Ling

**Affiliations:** Medical Officer to the Bristol Police


### The Bristol Medico-Chirurgical Journal

*" Scire est nescire, nisi id me*
*Scire alius sciret."*

SUMMER, 1938.

### PSYCHOLOGICAL FACTORS IN DISEASE.

BY

T. M. Ling, B.M., B.Ch., M.R.C.P.,
*Medical Officer to the Bristol Police.*

In all branches of Medicine, there is growing appreciation of the importance of emotional factors in disease, and contemporary literature shows this trend very clearly. As an example, Sir Maurice Cassidy has recently stated: " Beyond all question the most important advance in medical progress of recent years has been the recognition that psychological factors play an overwhelming part in the causation of invalidism, taken as a whole. It is now known that in panel practice the commonest diagnoses are 'debility,' 'dyspepsia,' 'gastritis,' 'anæmia,' 'neuralgia,' 'tachycardia,' and so on. And it is also known that the great majority of patients who are so diagnosed are really the victims of some psychological disorder, usually an anxiety state or a conversion hysteria. Moreover, this state of affairs is not confined to the hospital class of patient. These disorders are even more prevalent among the well-to-do classes. They constitute, for example, 30 per cent. of the cases seen in consulting cardiological practice."

This challenging statement from one of the leading members of the profession is really an indication that the orientation of medicine is changing from the materialism of the nineteenth century, and this change is intimately concerned with our conceptions of illness. In reality illness is not an entity, but rather a reaction to the forces of environment. Our primitive ancestors appreciated the fact that a severe accident would cause a broken leg ; later the effects of heat, cold and poisons were recognized, while in the last century the emphasis was chiefly laid on micro-organismal disease. Within the last thirty years it is generally recognized that food deficiencies must be added to these various extrinsic factors in the causation of illness, and now psychological disorders must be included in the list. They are in reality reactions in the "neurosis" system of the body, which is made up of the sympathetic system, the endocrines and the optic thalamus working in concert.

This neurosis system is essentially a defence mechanism against adverse factors in the emotional environment of the individual, and these factors have increased in importance of recent years for the following reasons :—

1. *The Insecurity of our Civilization.*—Contemporary literature is full of references to racial and ideological problems, and the increased facilities for the dissemination of news undoubtedly bring these problems to the notice of the ordinary citizen to an extent that has never happened in the past.

2. *Urbanization.*—Our civilization to-day is based on the town as the unit, and the majority of our patients are leading lives totally at variance with those led in the past. As a result we have problems of small families, overcrowding, and lack of opportunities for physical recreation that must play an important part in the development of the individual. In addition, the rationalization of industry has resulted in a large proportion of the population working under comparatively rigid conditions and under the authority of other people, with the result that personal relationships play a prominent part in the individual's daily life. The lack of creativeness and the sense of dependency that are so frequently associated with contemporary employment are all in conflict with our instinctive urges.

3. *The Fear of Disease.*—On all sides our patients are being assailed with cleverly-worded advertisements stressing possible dangers to health and offering panaceas after they have succeeded in stimulating the sense of fear that is dormant in everybody. The majority of people are literate rather than educated, and are not in a position to criticize the slogans they encounter on all sides.

4. *Decline of Religious Values.*—This important aspect is self-evident in the contemporary world and must result in a very different attitude to problems as they arise in patients' daily lives. At first sight these subjects may appear to be extraneous to our concepts of medicine and the causation of disease, but they are all capable of stimulating a sense of fear and insecurity with their attendant effects on the sympathetic system. Now all people of necessity become emotional when they encounter a difficulty, but the mature individual faces the problem objectively and maintains his functional integrity. Unfortunately the majority of our patients are in varying ways immature in that they have never quite grown up and still cling to many childish values that were helpful in early days, but are a hindrance in adult life. These immature people try to escape from their difficulties and this flight is associated with a sense of fear, together with resentment against the situation in question. These emotional disturbances are expressed in effects on viscera innervated by the autonomic nervous system, especially by its sympathetic division. It has been shown by Langley, Cannon, and others, that stimulation of this division causes changes throughout the whole body. Thus the hair stands on end, the digestive secretions are inhibited, the heart beats more quickly, the blood-pressure is raised, and the rectum and bladder are stimulated. These profound disturbances of the body equilibrium are in reality atavistic survivals from our primitive ancestors, as they enabled them to fight or to flee in the face of a sudden danger. The mechanism is of course still stimulated to-day by sudden peril, as for instance in a possible motor accident, but if the stimulation is protracted, the effects on the whole system must of necessity be very destructive. Although all viscera are influenced in this way by emotion, it frequently happens that one part may be picked out and its rhythm completely altered, with the result that the patient becomes conscious of a mechanism that under healthy conditions works entirely without his knowledge. The reasons for this are various ; in some cases the organ may be hereditarily inferior, as occurs in cases of asthma with a strong family history ; in other cases part of the body may have particular significance for the person, owing to early training and parental example. Thus prolonged stresses of an emotional type will produce tachycardia in one person, a flatulent dyspepsia or a duodenal ulcer in the next, and vaginismus in the third.

### Methods of Determining Ætiology.

Once it is agreed that psychic factors in the patient's background are intimately concerned with his presenting symptoms, it is essential that a complete history of the patient should be obtained so that the relative importance of the various factors can be accurately assessed. Inquiries must be made regarding occupation and the feelings the person has about his work, domestic life (particularly with regard to sex) and the attitude that the patient has to his particular illness. The latter is always important, as the fear of cancer or any disease from which some other member of the family may have suffered frequently complicates the clinical picture. This information can only be obtained by a specific technique in which all semblance of criticism is removed. Many people are frightened of what we think of them and will immediately avoid any particular aspect of their past if they think it is going to produce a sense of shame or disgust in the listener. This objectivity is essential in understanding all psycho-neurotic disturbances and the same attitude must be displayed when information is obtained from relations or parents.

### Relation of Organic to Functional Disorder.

From what has been said above regarding the profound influence of the sympathetic system on all organic function, it follows that organic and functional changes are always present in varying degrees in every case. An individual who is suffering from what at first sight appears to be a straightforward organic lesion is at the same time being influenced by his own feelings and fears about his presenting symptoms; while, conversely, the alteration in rhythm and function that follows any prolonged sympathetic disturbance is bound to result in structural change. The nineteenth-century differentiation of disease into organic and functional is therefore out of date and to-day the problem is one of deciding the relative proportions of each aspect that is present in any given case.

### Characteristics of Psycho-Neurotic Illness.

Disturbances of the emotional self are not fully understood by the conscious mind, while the influence of the latter is particularly important in all cases where the functional element predominates. Although a great deal appears in the popular press to-day about the sub-conscious mind, it should be realized that there is no sharp demarcation between consciousness and unconsciousness. One merges gradually into the other. It is now generally agreed that all past memories and experiences are, so to speak, stored away and inevitably influence our judgement and behaviour in any current situation. This state of affairs has been frequently compared to an iceberg of which only about one-eighth is visible above the surface, while the large bulk that is out of sight influences continuously the projecting portion. Although subconscious influences play a very important part in many functional disorders, at least half of these cases are capable of explanation and treatment on a conscious level.

Another important aspect of the problem which must be mentioned is the question of conflict. In all cases of psycho-neurotic illness there is a varying degree of conflict between the acquired self-regard of the individual, the "super-ego" of Freud and the instinctive drives of reproduction and self-preservation. The self-regard of the individual is dependent on early training and parental example, while our fundamental instincts are essentially hereditary and concerned with the preservation of the race. These two instincts may frequently be challenged on the conscious basis and conflict will result if the individual is unable to deal with the problem to his own satisfaction. Any state of conflict, whether conscious or unconscious, of necessity uses up a great deal of energy; hence the easy fatigability of the psycho-neurotic.

Although many cases of functional disorder can be adequately understood and treated on the conscious level, it is generally recognized that all such individuals have been the subject of conflicting sentiments in their earlier days, and that these have been rekindled by the present situation. These sentiments are essentially concerned with the relationship of the individual to his parents and home situation, and the seeds of adult emotional problems are always laid in the early years of life.

The treatment of cases which are predominantly emotional in origin is both preventive and curative. Under the former heading must be considered the "iatrogenic" or doctor-created diseases. There is no doubt that many conditions arise through faulty handling at the beginning. Advice is sought by patients primarily because they are anxious and the alleviation of this anxiety forms an integral part of the treatment, irrespective of the underlying pathology. In some cases difficulties may arise because insufficient time is given to the case. Fear arises in the doctor's mind that he may have perhaps missed something and fear is one of the most infectious of conditions. In addition, the patient feels that the case cannot be fully understood in the absence of his own views on the subject. This latter point is especially important, as the fear of such conditions as cancer, tuberculosis, heart-disease and lunacy is extremely prevalent. In the absence of a sympathetic listener the patient keeps these ideas to himself and the state of indecision that results in his own mind adds a further complication to the picture.

On the curative side it is essential to assess the relative extent of physical and psychical disorder. As mentioned above, both elements are present in every case and one of necessity influences the other. In what may be called mixed cases, it is very advisable to draw the patient's attention to the emotional factors that may be present, even if physical treatment is indicated at the beginning.

The particular type of psychological treatment that is indicated in any particular case varies according to the age, intelligence, type of personality of the patient, and the time available. A detailed history is in itself a valuable therapeutic measure, especially in cases that are sufficiently intelligent to understand how somatic symptoms may arise from emotional causes. Some cases are best dealt with by straight persuasion. This method was highly developed by Dejerine and is very efficacious with suggestible people of limited intelligence. Suggestion is of course an integral part of all medical work irrespective of the type of treatment being given. It is extremely valuable in some psycho-neurotic disorders, and can be reinforced by light hypnosis.

Where the problem is due to a conflict arising from subconscious causes, some form of analysis is essential for an adequate cure. The prime object of this approach is to help the patients to understand themselves in the light of their past experiences and behaviour. It is only applicable to people with a reasonable degree of intelligence and not over fifty years of age. In practice it is of course combined with re-education which enables the patient to deal with his current problems on a more objective basis.

### SUMMARY.

Psychological problems in medical and surgical work are much commoner than is generally appreciated ; approximately 30 per cent. of all cases of illness fall into this group.

Emotional conditions are appreciated and registered by the "neurosis" system, which consists of the sympathetic nervous system, the endocrines and the optic thalamus working in unison. As a result, somatic manifestations may occur in any part of the body.

The treatment is both preventive and curative. In all cases a full history should be taken, which will throw light on the social, marital and occupational problems in the patient's background. The type of psychological treatment depends on the age and intelligence of the patient rather than on the presenting symptoms. Persuasion coupled with an explanation of the mechanism involved is frequently sufficient in milder conditions, while more deep-seated problems demand some form of analytic investigation combined with re-education. Knowledge of the subject has increased very greatly of recent years and the future will see an even bigger extension of this aspect of medicine.

